# The Protective Effect of *Limosilactobacillus fermentum* FZU501 Against Alcohol-Induced Liver Injury in Mice via Gut Microbiota–Liver Axis

**DOI:** 10.3390/foods14061054

**Published:** 2025-03-19

**Authors:** Zihua Liang, Shiyun Chen, Xiangchen Zhang, Jiayi Li, Weiling Guo, Li Ni, Xucong Lv

**Affiliations:** 1 Institute of Food Science and Technology, College of Biological Science and Technology, Fuzhou University, Fuzhou 350108, China; 220820089@fzu.edu.cn (Z.L.); 220820074@fzu.edu.cn (S.C.); 228527202@fzu.edu.cn (X.Z.); 230827090@fzu.edu.cn (J.L.); t24048@fzu.edu.cn (W.G.); nili@fzu.edu.cn (L.N.); 2 Food Nutrition and Health Research Center, School of Advanced Manufacturing, Fuzhou University, Jinjiang 362200, China

**Keywords:** probiotic intervention, alcoholic liver injury, metabolomics, oxidative stress, intestinal microbiota, food microorganisms

## Abstract

As a probiotic strain isolated from *Hongqu* rice wine (a traditional Chinese fermented food), *Limosilactobacillus fermentum* FZU501 (designated as Lf) demonstrates exceptional gastric acid and bile salt tolerance, showing potential application as a functional food. The aim of this study was to investigate the protective effect of dietary Lf intervention on alcohol-induced liver injury (ALI) in mice. The results demonstrated that oral administration of Lf effectively ameliorated alcohol-induced lipid metabolism disorders by reducing the serum levels of TC, TG and LDL-C and increasing the serum levels of HDL-C. In addition, oral administration of Lf effectively prevented alcohol-induced liver damage by increasing the hepatic activities of antioxidant enzymes (CAT, SOD, GSH-Px) and alcohol-metabolizing enzymes (ADH and ALDH). Interestingly, 16S amplicon sequencing showed that oral administration of Lf increased the number of *Prevotella*, *Lachnospiraceae*_NK4A136_group and *Lactobacillus*, but decreased the proportion of *Faecalibaculum*, *Adlercreutzia* and *Alistipes* in the intestines of mice that consumed excessive alcohol, which was highly associated with improved liver function. As revealed by liver untargeted metabolomics studies, oral Lf clearly changed liver metabolic profiles, with the signature biomarkers mainly involving purine metabolism, taurine metabolism, tryptophan, alanine, aspartic acid and glutamate metabolism, etc. Additionally, Lf intervention regulated liver gene transcription in over-drinking mice for cholesterol metabolism, bile acid metabolism, fatty acid β-oxidation, alcohol metabolism and oxidative stress. Taken together, the above research results provide solid scientific support for the biological activity of Lf in ameliorating alcohol-induced liver metabolism disorder and intestinal microbiota imbalance.

## 1. Introduction

As a kind of beverage produced by biological fermentation process, alcoholic beverages are widely circulated and consumed around the world, and their raw materials are mainly derived from the biological transformation process of grains or fruits [1]. This dietary tradition with a long history has been deeply integrated into the cultural system of many countries [2]. However, according to data released by the World Health Organization in 2024, 209 million people (3.7% of the world’s adult population) suffer from alcohol dependence [3]. Excessive intake leads to ethanol accumulation exceeding the liver’s metabolic capacity, leading to intestinal barrier dysfunction and alcoholic liver injury (ALI)—a major cause of cirrhosis and hepatocellular carcinoma worldwide [4]. The latest study pushes the boundaries of conventional knowledge by demonstrating a positive linear correlation between ethanol intake and all-cause mortality in a large-scale cohort analysis, meaning that there is no theoretical safe intake range [5,6]. Although some drugs, including corticosteroids and hexonofylline, have been used to treat acute respiratory infections, long-term use of these drugs can cause a range of side effects [7]. This dilemma highlights the urgent need to highlight the public health hazards of alcohol abuse and the urgency of public health education and the development of safe dietary intervention strategies to reduce the harm of alcohol while respecting cultural traditions.

As research continues to deepen, the concept of “gut–liver” axis in the scientific community emerges with tremendous impacts on the pathogenesis and treatment of ALI. Several studies have shown that excessive alcohol consumption destroys the balance of the intestinal microbiota and the integrity of the intestinal barrier, which is mainly characterized by the reduction in the abundance of beneficial bacteria, the loss of tight junction proteins, and the disappearance of goblet cells [8]. Disruption of the intestinal barrier increases the translocation of microbial harmful metabolites, leading to more lipopolysaccharides entering the portal circulation, triggering endotoxemia and stimulating an inflammatory response [9]. Probiotics ubiquitously present in traditional fermented foods demonstrate therapeutic potential for intestinal health, with proven efficacy in pH reduction, microbiota homeostasis modulation (upregulating *Bifidobacterium* spp.), and bile acid metabolic regulation under appropriate intake conditions [10]. The effectiveness of oral administration of probiotics in promoting alcohol metabolism and combating ALI has been previously demonstrated [11]. As an important probiotic, *Limosilactobacillus fermentum* (formerly *Lactobacillus fermentum*) has been included in China’s Edible Microorganism List and is recognized as “Generally Recognized as Safe (GRAS)” by the U.S. Food and Drug Administration (FDA) [12]. According to literature reports, *Limosilactobacillus fermentum* exhibits broad application prospects in enhancing the nutritional value of food and improving flavor profiles. It also demonstrates multiple functional properties such as regulating gut microbiota, antioxidant effects, and hypoglycemic activity [13,14].

In recent years, the application of probiotics in the food industry has been expanding from the field of traditional fermented foods to the field of functional foods. A strain of *Limosilactobacillus fermentum* FZU501 (Lf) with strong gastric acid and bile salt tolerance was isolated from *Hongqu* rice wine, a traditional fermented food in China. However, the beneficial effects of oral Lf on intestinal microbiota and liver metabolism in ALI mice and its mechanism of action have not been fully elucidated. This study attempted to investigate the protective effect of oral Lf against ALI and its potential mechanism by high-throughput sequencing, non-targeted metabolomics, and RT-qPCR analysis. The research work is expected to provide a theoretical basis for developing new functional foods based on probiotics to alleviate health problems caused by excessive alcohol intake.

## 2. Materials and Methods

### 2.1. Materials and Reagents

*L. fermentum* FZU501 was strictly sorted from *Hongqu* wine by our research group in the early stage and stored in the refrigerator at −80 °C. The Man–Rogosa–Sharpe (MRS) broth medium was purchased from HuanKai Microbial Technology Co., Ltd. (Guangzhou, China). The silymarin was purchased from Sigma-Aldrich (St. Louis, MO, USA). The antioxidant and biochemical assay kits were provided by Solarbio Science & Technology Co., Ltd. (Beijing, China). Unless otherwise noted, all other reagents used in this study were obtained from Aladdin Biotech Co., Ltd. (Shanghai, China).

### 2.2. Preparation of Lf

The fermentation seeds were initially cultivated by incubating *L. fermentum* FZU501 in MRS broth at 37 °C for a period of 24 h. Afterward, the seed suspension from the *L. fermentum* FZU501 culture was collected, and a bacterial solution with an optical density of 0.6 to 0.8 was introduced into the MRS broth, and placed in a thermostatic incubator (37 °C, 24 h). After 24 h of cultivation, bacterial cells were collected and washed three times with sterile saline, and then re-suspended in sterile saline (adjusted to 5 × 10^9^ CFU/mL), which was used for future administrative purposes.

### 2.3. Animals and Experimental Design

Thirty-two Kunming mice (6-week-old, male) were obtained from HFK Biotech Co., Ltd. (Beijing, China), which did not carry specific pathogens. All mice were housed in the standard environment (temperature 22 ± 1 °C, humidity 50–55%, light/dark cycle for 12 h), with ad libitum water and food, and information on the diet of the mice is provided in Supplementary Tables S1 and S2. After the one-week acclimatization, the mice were randomly divided into four groups, namely Control (conventional feeding), Model (alcohol-induced liver injury model), PC (positive control, silymarin with clinically proven efficacy was selected as the intervention drug [15]) and Lf (Lf intervention). Mice of the Model, PC, and Lf groups received oral gavage of 50% alcohol solution (*v*/*v*, 7.5 mL/kg b.w.) daily, 4 h after the administration of 0.2 mL physiological saline, silymarin (100 mg/kg/day) and Lf (10^9^ CFU/day), respectively. Mice in the Control group were given an equivalent volume of physiological saline (the experimental feeding scheme of mice is shown in Supplementary Figure S1). The weight of each mouse was measured and documented weekly during the course of the experiment. Following 42 days of intervention, all mice were euthanized under anesthesia at 12 h after fast. The samples of fresh blood, liver, kidney, spleen and colon were collected into sterile centrifuge tubes. The serum was collected by centrifugation after blood samples were kept at room temperature for 30 min. Liver, spleen, kidney samples were weighed before diving. Partial liver tissue was placed in a liquid nitrogen tank and stored in a −80 °C refrigerator, and the relevant indexes were subsequently detected, while the others were transferred to 4% paraformaldehyde fixative. The mouse experimental procedures for this study met the ethical standards set by the Ethics Committee of the host institution (Approval No.: FZU-IFST-2022-001).

### 2.4. Serum and Liver Biochemical Tests

The concentrations of serum total cholesterol (TC), triglyceride (TG), low-density lipoprotein cholesterol (LDL-C) and high-density lipoprotein cholesterol (HDL-C), aspartate aminotransferase (AST) and alanine aminotransferase (ALT) were detected using assay kits. In addition, the high-speed homogenizer was applied to homogenize the mix of partial liver tissue and sterile saline. The supernatant of each sample was collected after centrifugation (10,000× *g*, 4 °C, 10 min). The concentrations of catalase (CAT), superoxide dismutase (SOD), glutathione peroxidase (GSH-Px), ADH, and ALDH were measured using assay kits.

### 2.5. Histopathological Analysis of Liver and Colon

The liver and colon tissues of mice were fixed in 4% paraformaldehyde solution for more than 24 h. After dehydration and paraffin embedding, the liver and colon tissues were sliced and stained with hematoxylin–eosin (H&E), and the morphology of the tissues and cells was observed under a microscope.

### 2.6. Quantification of Fecal Short-Chain Fatty Acids (SCFAs)

The fecal SCFAs were extracted and quantified using a previously established method [16]. Briefly, feces were collected, freeze-dried, and weighed. Freeze-dried feces (50 mg) and saturated NaCl (500 μL) solution were mixed and stored (25 °C, 30 min). Then, 10 μL of hydrochloric acid was transferred to the sample solution and homogenized. After centrifugation (12,000× *g*, 4 °C, 10 min), the supernatant was transferred to 0.8 mL of absolute ether for SCFAs extraction and 0.2 g of anhydrous Na_2_SO_4_ was added to remove trace water from the sample. After shaking and centrifugation, the SCFAs in the supernatant were collected and determined by Agilent 7890B gas chromatography.

### 2.7. Intestinal Microbiota Sequencing Analysis

Genomic DNA was extracted from feces using a test kit (MoBio, Calsbad, CA, USA) The high-throughput sequencing technology was applied by the Illumina MiSeq platform (Shanghai Majorbio Co., Ltd., Shanghai, China) to sequence the V3–V4 hypervariable region of 16S rDNA. QIIME 2 software (Ver. 2019.7) was applied to treat the raw data, and the filtered sequences with a 97% identity threshold were clustered into amplicon sequence variants (ASVs). Subsequently, the representative sequences from each ASV were selected and annotated using the GreenGenes database (Ver. 13.8). Among the Model group and other (Control, PC, and Lf) groups, the significantly different bacterial phylotypes were screened through STAMP software (version 2.1.3) based on the Welsh *t*-test. The relationships between key intestinal bacterial phylotypes and biochemical parameters were computed and visualized using the pheatmap package through R software (Ver. 4.1.2) and Cytoscape (Ver. 3.9.0), respectively.

### 2.8. Liver Metabolomics Analysis

The liver tissue (50 mg) was thoroughly mixed with 500 µL organic solution (acetonitrile: methanol: water = 2:2:1), and the liver tissue homogenate was prepared by an ultrasonic crusher. The supernatant was collected by centrifugation and dried by nitrogen blowing evaporation. The dried samples were re-suspended with 200 µL acetonitrile (50%, *v*/*v*) and centrifuged at 4 °C and 12,000 r/min for 15 min to remove insoluble matter. In order to ensure the purity of the sample, the supernatant was further filtered by a 0.22 µm organic filter membrane to prepare the sample to be detected. Then, an ultra-high performance liquid chromatography–QTOF electrospray ionization mass spectrometer (1290 Infinity UPLC-6530 QTOF electrospray ionization MS system (Agilent, Santa Clara, CA, USA) with an UPLC-BEH amide column (2.1 × 100 mm, 1.7 µm, Waters) was used for detection. The original data were imported into MPP software through CEF transformation for analysis (Agilent, CA, USA). Principal component analysis (PCA) and orthogonal partial least squares discriminant analysis (OPLS-DA) of liver metabolic profiling were carried out by SIMCA-14.1 software (UMETRICS, Umetrics, Sweden). Based on the analysis model of OPLS-DA, an s-loading plot was applied to screen liver biomarkers between the Model and Lf groups, with the screening criteria of VIP > 1.0 and *p* < 0.05. Enrichment of metabolic pathways of liver biomarkers was performed using the MetaboAnalyst 5.0 (https://www.metaboanalyst.ca, accessed on 11 April 2023).

### 2.9. Quantitative Gene Transcription Assay

Total RNA was extracted from liver tissues using the miRNeasy Mini Kit (Vazyme, Nanjing, China), and the mRNA was reverse-transcribed into cDNA using the PrimeScriptTM RT reverse transcription kit (Takara, Beijing, China). Mouse 18S rDNA was used as the internal reference gene. PCR was performed under the operation requirements of the Step One Plus real-time PCR system and Tli RNaseH Plus kit with primers targeting specific genes (Supplementary Table S3), and the expression levels of liver function-related genes were analyzed by the 2^−ΔΔCt^ method.

### 2.10. Statistical Analysis

Data from different experimental groups were analyzed statistically between groups using the GraphPad Prism software (Ver. 9.5). One-way ANOVA using SPSS (v 23.0) was used to assess the test results’ statistical significances, which were as follows: * *p* < 0.05 versus the Control group, ^#^ *p* < 0.05 versus the Model group; ** *p* < 0.01 versus the Control group, and ^##^ *p* < 0.01 versus the Model group.

## 3. Results and Discussion

### 3.1. Effects of Lf on Body Weight and Organ Indexes

**Figure 1:**
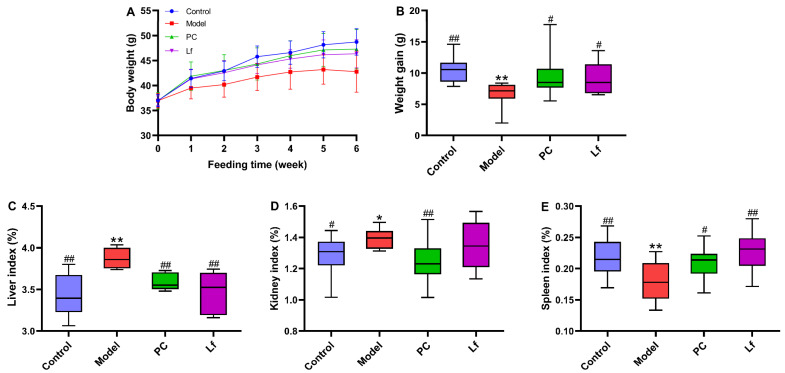
Effects of oral Lf on body weight and organ index in mice with ALI. (**A**) Growth curve; (**B**) weight gain; (**C**) liver index; (**D**) kidney index; (**E**) spleen index. Compared with the Control group, 0.01 < * *p* < 0.05 and ** *p* < 0.01; compared with the Model group, 0.01 < ^#^ *p* < 0.05 and ^##^ *p* < 0.01.

At the beginning of the experiment, the average body weight of the mice in each group was similar, and there was no significant difference (Figure 1). The body weight of mice in the control group increased continuously during the whole experiment, which was obviously higher than that in the experimental groups treated with alcohol (including Model group, PC group and Lf group), indicating that long-term alcohol consumption may destroy the metabolic function of mice, which is consistent with the findings of a previous study [17]. Our study found that both the silymarin and Lf interventions were effective in preventing anomalous weight decline due to alcohol ingestion (*p* < 0.05). Compared with the Control group, the liver and kidney indexes in the Model group abnormally increased (*p* < 0.05), and the spleen indexes abnormally decreased (*p* < 0.01), indicating that excessive drinking caused swelling of the liver and kidney and atrophy of the spleen. Interestingly, oral Lf improved the abnormal elevation of liver index and abnormal reduction in spleen index (*p* < 0.05). These results indicate that Lf can inhibit phenotypic abnormality of ALI mice to a certain extent.

### 3.2. Effects of Lf on Serum Biochemical Parameters

**Figure 2:**
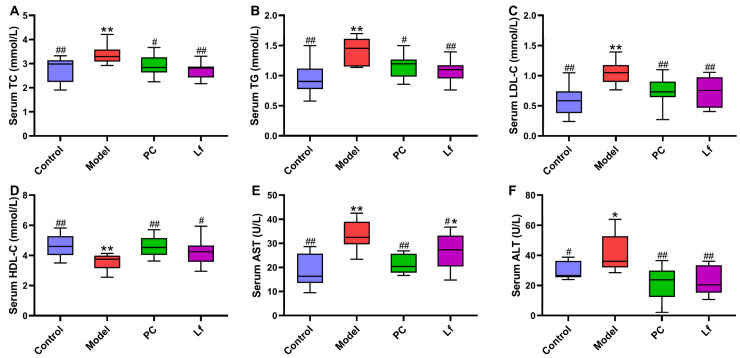
Effects of oral Lf on the serum biochemical parameters in mice with ALI. (**A**) TC; (**B**) TG; (**C**) LDL-C; (**D**) HDL-C; (**E**) AST; (**F**) ALT. Compared with the Control group, 0.01 < * *p* < 0.05 and ** *p* < 0.01; compared with the Model group, 0.01 < ^#^ *p* < 0.05 and ^##^ *p* < 0.01.

At the end of the experiment, serum biochemical indexes of mice in each group were measured, and the results are shown in Figure 2. Compared with the Control group, the contents of TC, TG and LDL-C in the serum of the model group were significantly increased (*p* < 0.05), but the content of HDL-C in serum was significantly decreased (*p* < 0.01). According to a previous study, excessive alcohol consumption could destroy the body’s lipid metabolism, characterized by elevated serum levels of TC, TG and LDL-C [18], which is consistent with the results of this study. However, oral administration of both silymarin and Lf significantly improved lipid metabolism disorders in mice. The activity of serum ALT and AST is an important index to evaluate liver function, which reflects the damage degree of liver cell membrane and mitochondria, respectively [19]. After six weeks of silymarin treatment, the serum ALT and AST activities were significantly reduced in mice with ALI (*p* < 0.05). However, Lf intervention only significantly reduced the serum ALT activity in mice with ALI (*p* < 0.05). In conclusion, Lf interventions effectively inhibit lipid metabolism disorder and alleviate hepatocyte injury in mice with ALI.

### 3.3. Effect of Lf on Alcohol-Induced Liver Oxidative Stress

**Figure 3:**
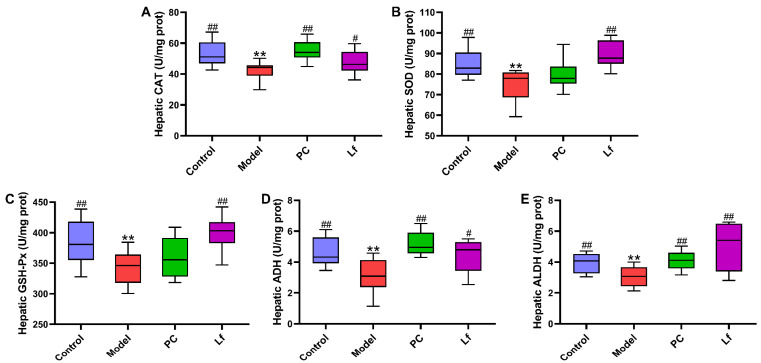
Effects of oral Lf on the hepatic biochemical parameters in mice with ALI. (**A**) CAT; (**B**) SOD; (**C**) GSH-Px; (**D**) ADH; (**E**) ALDH. Compared with the Control group, ** *p* < 0.01; compared with the Model group, 0.01 < ^#^ *p* < 0.05 and ^##^ *p* < 0.01.

Oxidative stress is one of the main reasons for the occurrence and deterioration of ALI, which is caused by the destruction of the balance between oxidative and antioxidant systems [20]. As shown in Figure 3, excessive intake of alcohol significantly reduced the hepatic CAT, SOD, GSH-Px, and ADH activities (*p* < 0.05). However, Lf intervention effectively increased the hepatic CAT, SOD, GSH-Px, ADH and ALDH activities in mice with ALI (*p* < 0.05). Activated SOD and CAT activities prevent the accumulation of reactive oxygen species (ROS), which is beneficial for reducing severe injury to DNA, proteins and lipids, otherwise accelerating the development of some diseases [21]. The decomposition products (hydrogen peroxide) of ROS were degraded into water and oxygen by elevating GSH-Px activity [22]. In addition, ADH and ALDH act as the most important enzymes that prevent alcohol accumulation in the body. The elevation of ADH can promote the conversion of ethanol to acetaldehyde, which then oxidizes into acetic acid by ALDH. Afterwards, acetic acid can be quickly eliminated from the body, thus reducing the damage of alcohol to the body [7]. In summary, Lf can reduce oxidative stress in mice, and facilitate the alcohol metabolism, thereby potentially alleviating the symptoms of ALI in mice.

### 3.4. Effects of Lf on Fecal SCFAs Levels

**Figure 4:**
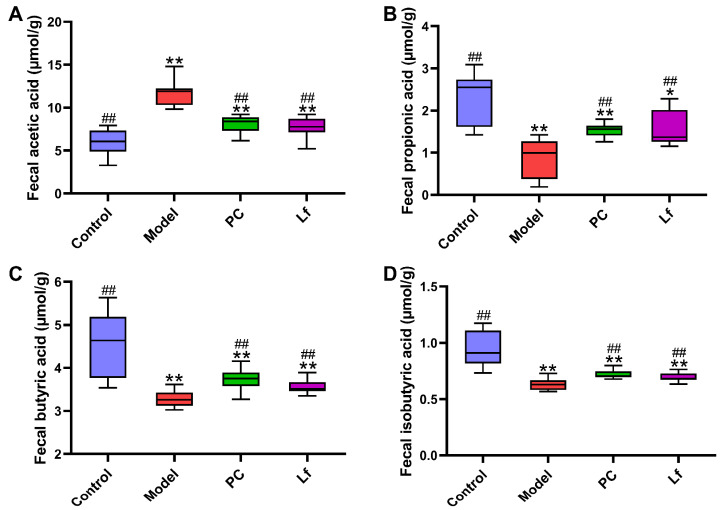
Effects of oral Lf on fecal SCFAs concentration in mice with ALI. (**A**) Acetic acid; (**B**) propionic acid; (**C**) n-butyric acid; (**D**) isobutyric acids. Compared with the Control group, 0.01 < * *p* < 0.05 and ** *p* < 0.01; compared with the Model group, ^##^ *p* < 0.01.

SCFAs mainly stem from dietary fiber by intestinal microbiota, which provide energy for ileal cells and inhibit the growth of harmful microorganisms [23]. In this study, the levels of four SCFAs in each mouse among all groups were detected by gas chromatography (Figure 4). Compared with the Control group, excessive consumption of alcohol significantly elevated the fecal acetic acid level (*p* < 0.01), and obviously reduced the fecal ropionic, n-butyric and isobutyric acid levels (*p* < 0.01). We speculated that the elevated acetic acid levels in the Model group of mice may be due to excessive alcohol intake, which leads to large amounts of its conversion to acetic acids. In addition, it has been reported that n-butyric acid and propionic acid are considered as preferred energy sources for intestinal epithelial cells, helping to promote the integrity and function of the intestinal wall and indirectly promoting the absorption of nutrients [24]. Notably, the levels of propionic acids, n-butyric acids and isobutyric acids were significantly lower in the Lf group than in the model group (*p* < 0.05). On the other hand, n-butyric acids and propionic acids help to reduce the risk of hyperlipidemia and regulate the expression of mucin and tight junction proteins, thus avoiding the occurrence of intestinal leakage caused by alcohol overdose [25]. Isobutyric acids are confirmed to enhance insulin-stimulated glucose uptake in rat adipocytes, which is helpful to improve insulin sensitivity in patients with metabolic disorders [26]. Therefore, we speculated that the mitigative effects of Lf on ALI may be achieved in part by regulating gut microbial composition and its metabolites.

### 3.5. Effects of Lf on Histopathological Features of Liver and Intestine

**Figure 5:**
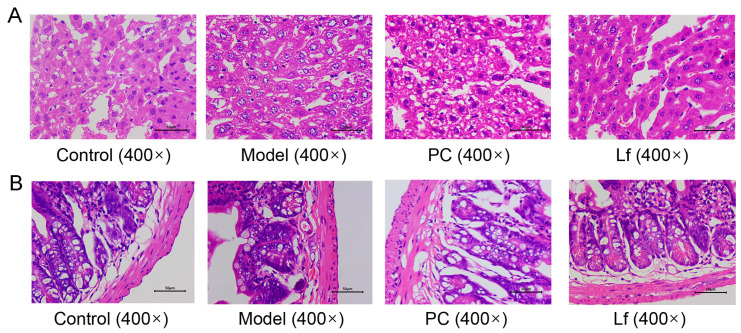
Effects of oral Lf on the histopathological features of liver (**A**) and intestine (**B**) in mice with ALI.

The histological examination of liver and colon tissues from each group was observed by employing H&E-stained sections (Figure 5A). The liver structure of mice in the Control group exhibited a complete and clear hepatic lobular structure, with hepatocytes regularly arranged in a radial pattern extending from the central vein to the periphery, with a relatively uniform hepatocyte size and no degeneration or necrosis, and with nuclei in the center of the cells. In contrast, long-term alcohol exposure resulted in liposathia in mouse hepatocytes, swelling of most of the nuclei, dense vacuoles of varying sizes in the cytoplasm, and destruction of liver structure. Nevertheless, the administration of silymarin and Lf resulted in a restoration of the structural disorder, inflammatory cell infiltration, and blurring of cell boundaries. Furthermore, some studies suggested that long-time consumption of alcohol could destroy the intestinal barrier integrity, which promotes the development of metabolic disease [9,27]. As shown in Figure 5B, the colon of Model group mice exhibited the loss of mucosal architecture, decrease in stem cells and goblet cells, and the destruction of crypt cells. But the degree of colonic lesions of the mice treated with silymarin and Lf was reversed, and the main manifestations were the thickness of the colonic muscle and the arrangement of glands, which tended to be normal, and the epithelium and cup cell structure were restored. These results provided evidence that Lf has an improvement effect on liver and colon damage in mice with ALI.

### 3.6. Effects of Lf on the Composition of Intestinal Microbiome

**Figure 6:**
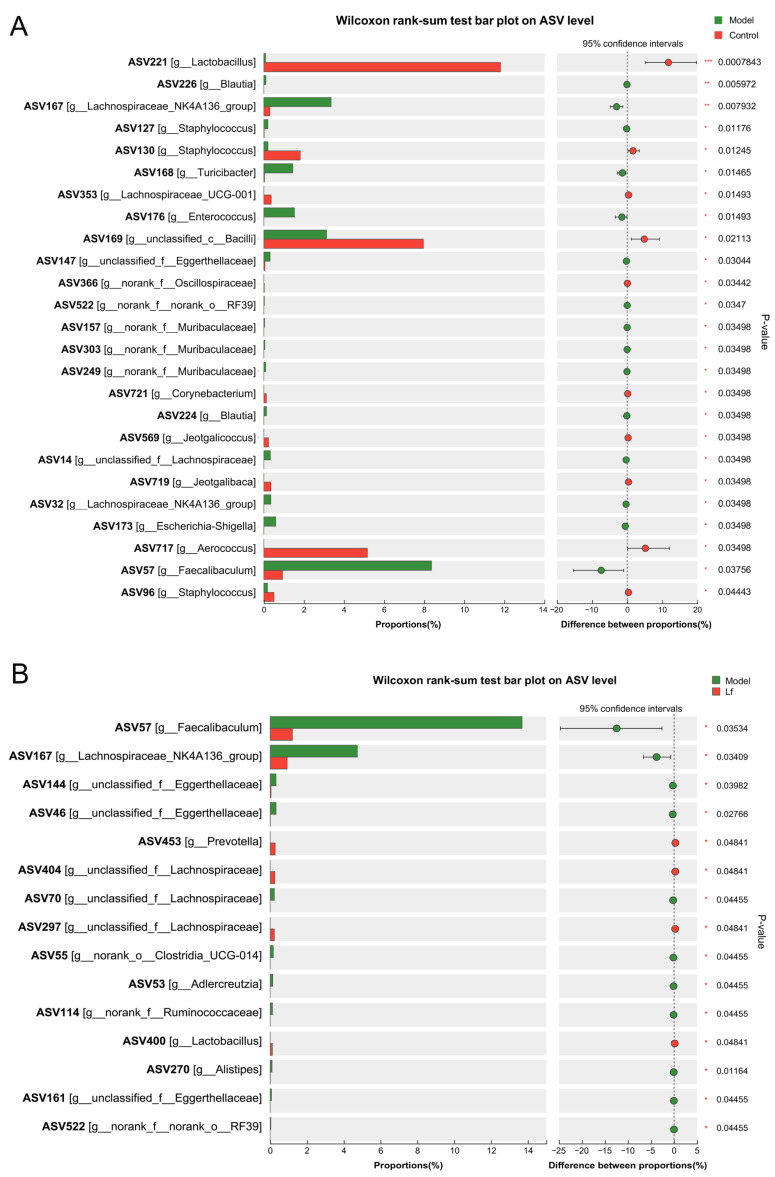
The differences in the relative abundances of intestinal bacterial genera between different experimental groups. (**A**) The Control group versus the Model group; (**B**) the Lf group versus the Model group.

The gut microbiome plays an important role in both host health and disease through secreting a series of metabolites. Previous studies have shown that alcohol can contribute to the development and progression of ALI by altering the composition, quantity, and product of the gut microbiome, or by disrupting the intestinal wall barrier function and causing liver translocation [28]. Therefore, the effects of Lf on the composition of gut microbiota in mice with ALI at the genus level were revealed using 16S rDNA sequencing. Compared to the Control group, the Model group exhibited a significant increase in the proportion of *Blautia* (ASV226), *Lachnospiraceae*_NK4A136_group (ASV167), *Staphylococcus* (ASV127), *Turicibacter* (ASV168), *Enterococcus* (ASV176), *Escherichia-Shigella* (ASV173) and *Faecalibaculum* (ASV57) in the Model group, while the proportion of *Lactobacillus* (ASV221), *Staphylococcus* (ASV96 and ASV130), *Lachnospiraceae*_UCG-001 (ASV353), *Corynebacterium* (ASV721), *Jeotgalicoccus* (ASV569), *Jeotgalibaca* (ASV719) and *Aerococcus* (ASV717) was remarkably reduced (Figure 6A). *Blautia* is a genus of anaerobic bacteria, which was reported to be positively correlated with serum ALT, AST, TG and TC [29]. *Staphylococcus* is a common and virulent pathogen in clinical practice, and infection can lead to abnormal liver function and accelerate destructive hepatic artery thrombosis [30]. *Turicibacter* is a sulfate-reducing bacterium closely associated with inflammatory bowel disease and is strongly associated with inflammation of the colon [31]. In addition, *Escherichia-Shigella* and *Enterococcus* are generally considered to be pro-inflammatory gut microbes [6]. Among them, *Enterococcus,* as a resident bacterium in the host, has been implicated in exacerbating ethanol-related liver disease by triggering hepatic inflammation and hepatocyte loss [32]. In contrast, *Lactobacillus* belongs to the phylum firmicutes that is part of the normal human gut microbiota, which could modulate the immune response to prevent the development of some diseases [33]. Overconsumption of alcohol may decrease the relative abundance of *Jeotgalicoccus*, which exacerbates ALI progression [34]. These findings indicate that excessive alcohol consumption may impair liver health by disrupting the balance of gut microbiota. However, the administration of Lf dramatically elevated the relative abundance of *Prevotella* (ASV453), g_unclassified_f_*Lachnospiraceae* (ASV404) and *Lactobacillus* (ASV400), but accidently decreased the relative abundance of *Faecalibaculum* (ASV57), *Lachnospiraceae* NK4A136_group (ASV167), unclassified_f_*Eggerthellaceae* (ASV46 and ASV144), *Adlercreutzia* (ASV53) and *Alistipes* (ASV270) in mice with excessive alcohol consumption (Figure 6B). *Prevotella,* a commensal obligately anaerobic Gram-negative bacterium, improves the symptom of type 2 diabetes by suppressing the inflammatory responses and reducing the blood glucose [35]. As a classic probiotic, *Lactobacillus* plays an important role in maintaining the intestinal barrier, suppressing oxidative stress, inhibiting TG synthesis and regulating bile acid homeostasis [36,37]. *Lachnospiraceae* facilitates 7α-dehydroxylation, converting cholic acid and chenodeoxycholic acid into secondary bile acids that helps maintain bile acid homeostasis [38]. In addition, *Prevotella* and *Lactobacillus* are well known as SCFA-producing bacteria, which influence the host metabolic functions and inflammatory response. Conversely, *Adlercreutzia* is a Gram-positive and obligately anaerobic bacterium, and it has been reported that its abundance is strongly associated with leanness and obesity [39,40]. *Alistipes* is a relatively new bacterial genus, which widely exists in mice with colitis [41]. Taken together, the results suggested that Lf intervention can effectively affect the intestinal microbiota composition of mice with ALI.

**Figure 7:**
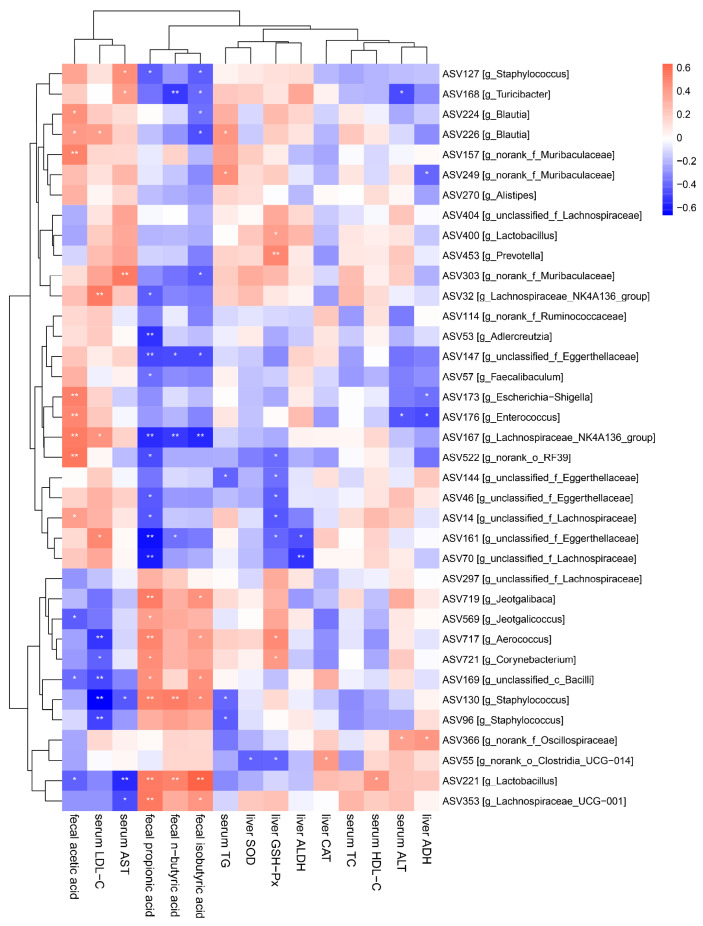
Correlation analysis between the biochemical parameters and the key intestinal microbial phylotypes.

It is the general consensus that gut microbiota is involved in regulating host physiological activities and signal transduction. Therefore, the potential correlations between gut microbiota and serum, and the liver biochemical indexes were explored using Spearman correlation analysis (Figure 7). The findings indicated that higher levels of fecal acetate, serum LDL-C and serum AST were positively linked to *Staphylococcus* (ASV127), *Turicibacter* (ASV168), *Blautia* (ASV224 and ASV226), norank_f_*Muribaculaceae* (ASV157 and ASV303), *Lachnospiraceae*_NK4A136_group (ASV32 and 167), *Enterococcus* (ASV176) and unclassifed_f_*Eggerthellaceae* (ASV161), but negatively associated with *Aerococcus* (ASV717), *Corynebacterium* (ASV721), unclassified_c_*Bacilli* (ASV169), *Staphylococcus* (ASV130 and ASV96), *Lactobacillus* (ASV221) and *Lachnospiraceae*_UCG-001 (ASV353). In addition, fecal n-butyrate, propionate and isobutyrate were negatively associated with *Staphylococcus* (ASV127), *Turicibacter* (ASV168), *Blautia* (ASV226), norank_f_*Muribaculaceae* (ASV303), *Lachnospiraceae*_NK4A136_group (ASV32, ASV53 and ASV167), *Adlercreutzia*, unclassified_f_*Eggerthellaceae* (ASV147), *Faecalibaculum* (ASV57), norank_o_RF39 (ASV522) and unclassified_f_*Eggerthellaceae* (ASV144, ASV46 and ASV161, unclassified_f_*Lachnospiraceae* (ASV14 and ASV70), but positively associated with *Jeotgalibaca* (ASV719), *Jeotgalicoccus* (ASV569), *Aerococcus* (ASV717), *Corynebacterium* (ASV721), unclassified_c_*Bacilli* (ASV169), *Staphylococcus* (ASV130), *Lactobacillu* (ASV221) and *Lachnospiraceae*_UCG-001 (ASV353). Therefore, combined with the phenomena observed above, Lf may play a positive role in improving liver damage caused by excessive drinking by regulating intestinal microbiota.

### 3.7. Effects of Lf on the Liver Metabonomic Profiling

**Figure 8:**
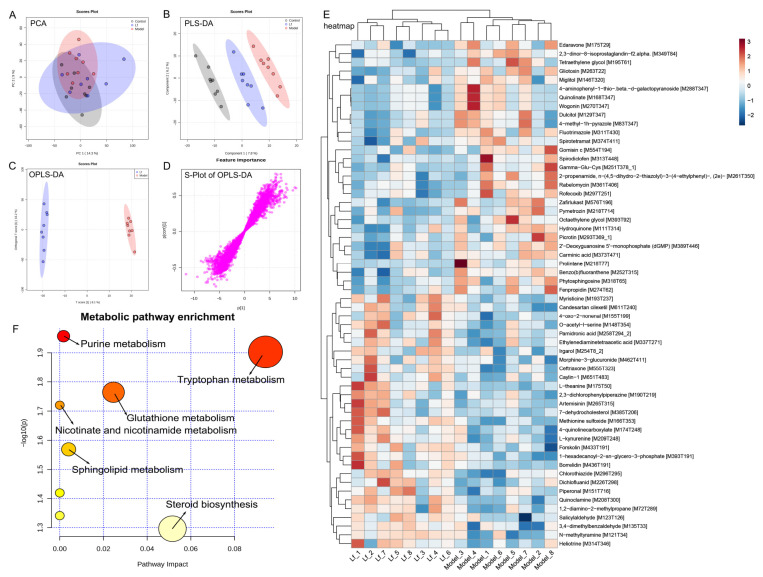
Liver metabolomic profiling by UPLC-QTOF/MS in the ESI+ model. (**A**) PCA score plot; (**B**) PLS-DA score plot; (**C**) OPLS-DA score plot; (**D**) S-loading plot; (**E**) intergroup differential metabolites; (**F**) metabolic pathway analysis.

**Figure 9:**
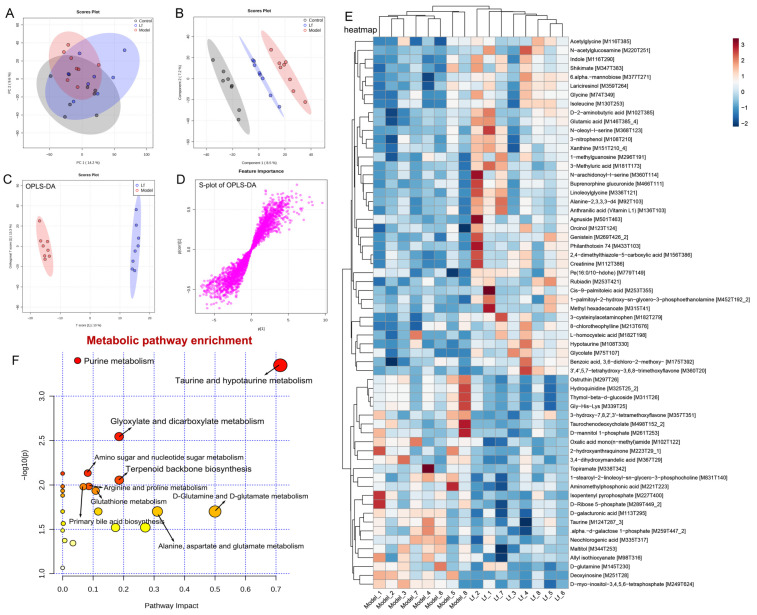
Liver metabolomic profiling by UPLC-QTOF/MS in the ESI− model. (**A**) PCA score plot; (**B**) PLS-DA score plot; (**C**) OPLS-DA score plot; (**D**) S-loading plot; (**E**) intergroup differential metabolites; (**F**) metabolic pathway analysis.

The liver serves as the primary site for the oxidative metabolism of ethanol. Prolonged and excessive alcohol consumption can lead to significant liver damage, contributing to the development of ALI [7]. This study explored the impact of Lf on liver metabolism in mice that experienced high levels of alcohol intake, utilizing untargeted metabolomics through UPLC-QTOF/MS. PCA and OPLS-DA were utilized to assess the differences in liver metabolic profiles among the Control, Model, and Lf groups (Figure 8A,B and Figure 9A,B). PCA is an unsupervised multivariate statistical method, which projects the data into the principal component space by linear transformation to visualize the similarities and differences in the overall sample [42]. Unlike PCA, PLS-DA and OPLS-DA are “supervised mode” multivariate statistical analysis methods that can exclude unnecessary intra-group errors and better analyze differences between groups [43]. The metabolic spectrum of the Control group was clearly separated from that in the Model group, suggesting that endogenous metabolites in mice liver changed by excessive consumption of alcohol. However, the Lf group showed partial distinction from the Model group, indicating that the disrupted metabolism could be partially reversed with Lf intervention. These results were further confirmed by OPLS-DA (Figure 8C and Figure 9C). In addition, the S-plots of OPLS-DA scoring plots were used to screen differences in liver metabolic profiles between the Lf and Model groups, based on VIP > 1.0 and *p* < 0.05 (Figure 8D and Figure 9D).

A total of 58 inter-group differential metabolites between the Model and Lf groups were identified in the positive ion (ESI+) mode. Among these, 29 differential metabolites showed decreased levels, and 29 differential metabolites increased after Lf intervention (Figure 8E). Gliotoxin is a potent mycotoxin, which can induce apoptosis through over-production of ROS, and mainly damage and kill microglial cells, astrocytes, and oligodendrocytes [44]. Quinolinate is an endogenous neurotoxin; long-term exposure to the environment containing quinoline can lead to the occurrence and development of tumors. Moreover, the liver also shows sensitivity to it; in other words, quinoline may poison animals and human liver parts [45]. Benzo[b] fluoranthene is one of the main compounds of polycyclic aromatic hydrocarbons (PAHs); in addition to the recognized carcinogenicity, it also has a variety of toxicity such as genetics, reproduction, etc.; in vivo, the liver can metabolize PAHs into phenols, quinones, and epoxides. During the process, it can generate a great quantity of ROS, and if not scavenged in a timely manner, it will result in oxidative damage to the liver [46]. Phytosphingosine is a potential diagnostic biomarker of liver cirrhosis and hepatocellular carcinoma [47]. The oral administration of Lf significantly reduced the hepatic gliotoxin, quinolinate, benzo[b]fluoranthene and phytosphingosine levels in mice with excessive alcohol consumption. However, it was also observed that this administration significantly elevated the hepatic candesartan cilexetil, L-theanine, artemisinin, L-kynurenine, methionine sulfoxide and borrelidin levels. Among them, candesartan cilexetil is a selective angiotensin II type I receptor antagonist, which has a series of physiological properties, such as anti-inflammatory, antioxidant, and anti-cancer effects [48]. L-theanine is a nonprotein amino acid that promotes the browning of white adipose tissue by regulating the AMPK/α-Ketoglutarate/Prdm16 axis and alleviates oxidative stress and inflammation by mediating the p38/MAPK signaling pathway [49]. Artemisinin is a sesquiterpene lactone compound, which not only has anti-malaria effects, but also has strong anti-inflammatory and antioxidant activities, which can reduce the expression of inflammatory factors and alleviate the damage of liver inflammation [50]. L-kynurenine is a pivotal metabolite of tryptophan degradation, which has been shown to possess the capacity to inhibit colitis by stimulating Treg cells [51]. Borrelidin is an 18-membered macrolide polyketide that inhibits the differentiation of adipocyte by regulating GATA-3 expression [52]. Furthermore, pathway enrichment analysis of differential metabolites (ESI+) was analyzed by the MetaboAnalyst 5.0 platform. A total of six metabolic pathways were identified, including purine metabolism, tryptophan metabolism, glutathione metabolism, nicotinate and nicotinamide metabolism, sphingolipid metabolism and steroid biosynthesis (Figure 8F).

In the negative ion (ESI-) mode, a total of 62 differential metabolites between the Model and Lf groups were collected, including the reduction in 24, and the increase in 38 differential metabolites in mice with ALI after Lf intervention (Figure 9E). Among these, N-acetylglucosamine has a strong avidity for vimentin and desmin on cell surfaces, and may be useful for the stable and accurate diagnosis and therapy of liver fibrosis [53]. Indole stems from the tryptophan metabolism, which was reported to mitigate the inflammatory responses by activating the aryl hydrocarbon receptor (AhR) and suppressing the activation of the NF-κB signalling pathway in mice with excessive alcohol intake [54]. Glycine was demonstrated to ameliorate LPS-induced apoptosis, reducing the infiltration of inflammatory cells [55]. Glutamic acid is a precursor of glutathione, which is responsible for the anti-oxidative response inside the body, and its supplementation can contribute to the biosynthesis of glutathione, and hepatic glutathione metabolic pathways play an important role in the protection of the liver against oxidative stress, detoxification, and the regulation of cell apoptosis [56]. In present study, the Lf administration of mice with excessive alcohol consumption resulted in a remarkable elevation of hepatic N-acetylglucosamine, indole, glycine and glutamic acid contents, while significantly reducing the hepatic allyl isothiocyanate, taurochenodeoxycholate and topiramate contents. Allyl isothiocyanate has been reported to possess carcinogenic activity [57]. Taurochenodeoxycholate is a key metabolite that affects lipid accumulation and inflammation in the liver [58]. Topiramate is a conventional drug used to treat epilepsy, and its side effects can lead to liver and kidney damage, behavioral abnormalities, peripheral nerve damage, mood abnormalities, and impaired cognitive functioning [59]. In the ESI- mode, the metabolic pathways that underwent the most significant changes as a result of the Lf intervention included purine metabolism, taurine and hypotaurine metabolism, glyoxylate and dicarboxylate metabolism, and amino sugar metabolism. In addition, the intervention resulted in significant alterations in nucleotide sugar metabolism, terpenoid backbone biosynthesis, arginine and proline metabolism, primary bile acid biosynthesis, D-glutamineand, etc. (Figure 9F).

### 3.8. Effects of Lf on Liver Gene Transcription Levels

**Figure 10:**
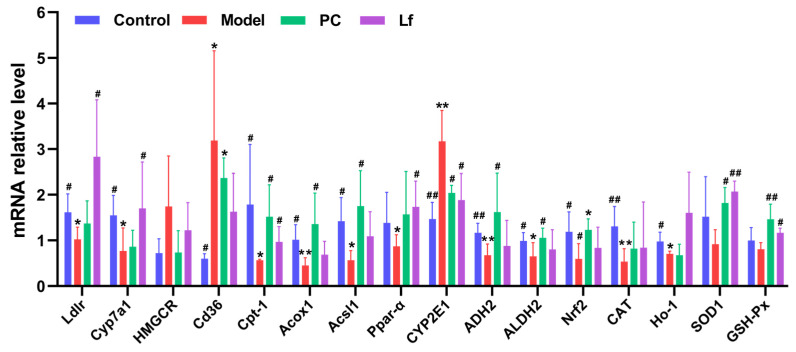
Differences among groups in the mRNA levels of hepatic genes in mice with ALI. Compared with the Control group, 0.01 < * *p* < 0.05 and ** *p* < 0.01; compared with the Model group, 0.01 < ^#^ *p* < 0.05 and ^##^ *p* < 0.01.

To deeply explore the potential mechanism of Lf in ALI, mRNA expression levels of genes related to cholesterol metabolism, bile acid metabolism, lipid metabolism, alcohol metabolism and oxidative stress were analyzed by RT-qPCR (Figure 10). In comparison to the Control group, the hepatic expression levels of *Ldlr* and *Cyp7a1* were significantly decreased in the Model group (*p* < 0.05), while the expression level of hepatic *HMGCR* was somehow increased in the Model group (*p* > 0.05). In the blood circulation of the body, LDL-C combines with low-density lipoprotein receptor (LDLR) on the surface of liver cells to form a complex, which is transferred to the lysosome through cellular endocytosis, and then LDL-C is digested in the lysosome, while LDLR is recycled back to the surface of liver cells to further bind to LDL-C in order to reduce LDL-C in the body [34]. Therefore, the up-regulation of *Ldlr* gene expression can improve cholesterol metabolism, and the research results are consistent with Figure 2C. In contrast, the HMG-CoA reductase encoded by the *HMGCR* gene is a key rate-limiting enzyme in cholesterol biosynthesis, responsible for converting HMG-CoA to mevalonate, and when the *HMGCR* gene is overexpressed, the activity of HMG-CoA reductase increases, resulting in accelerated cholesterol synthesis pathways and elevated cholesterol levels [60]. In addition, Cyp7a1 serves as the key enzyme regulating bile acid synthesis, responsible for metabolizing more than half of the hepatic TC [61]. Lf intervention significantly up-regulated the expression of *Ldlr* and *Cyp7a1* in mice with ALI (*p* < 0.05), but slightly down-regulated the expression of *HMGCR* (*p* > 0.05), suggesting that Lf has a favorable ameliorative effect on liver cholesterol and bile acid metabolism in mice with ALI. In addition, previous studies have confirmed that excessive alcohol consumption leads to oxidative stress, which accelerates the process of ALI [62]. This study also found that excessive alcohol consumption significantly promoted the expression of liver adipogenesis gene *CD36* (*p* < 0.05), while simultaneously inhibiting the expression of liver fatty acid oxidation genes *Cpt-1*, *Acox1*, *Acsl1* and *Ppar-α* (*p* < 0.05). Among these, CD36 is extensively distributed in liver, fat and small intestine, and is considered to play an important role in the absorption of fatty acids. Elevated *CD36* gene expression promotes fatty acid uptake, lipid accumulation and disruption of glycolipid metabolism, ultimately leading to oxidative stress and inflammation in the live [63]. However, the β-oxidation of fatty acid oxidation is controlled by Cpt-1 and Acox1, which can promote the lipolysis of cells. Acsl1 plays an important role in the extension of the carbon chain of fatty acids, and Acsl1 can activate fatty acids into fatty acyl-coA, and enters the mitochondria for β-oxidation [64]. In addition, Ppar-α is a vital target that regulates the oxidation of fatty acid in mitochondria, and activates the expression of *ACOX1* to promote the breakdown of fatty acids, thereby reversing hepatocyte steatosis [65]. In this study, Lf intervention significantly reversed the change in these genes expression induced by excessive alcohol consumption, indicating that Lf treatment can reduce the uptake and transport of lipids in mice with ALI.

The influence of Lf on alcohol metabolism and oxidative stress in mice with ALI was investigated. Compared with the Control group, the expression level of hepatic *CYP2E1* was significantly increased in the Model group (*p* < 0.01), and the expression level of hepatic *ADH2* and *ALDH2* was obviously reduced (*p* < 0.05). Among them, long-term alcohol consumption induces an increase in *CYP2E1*, which promotes the oxidation and clearance of alcohol in the liver. Catalyzed by higher *CYP2E1* gene expression, ethanol oxidizes faster and produces more acetaldehyde and reactive oxygen species, leading to oxidative stress of hepatocytes and excessive production of acetaldehyde, damaging hepatocyte mitochondria and promoting hepatocyte apoptosis [66]. Both ADH and ALDH have a dimeric structure, and their sub-units can be encoded by *ADH2* and *ALDH2* genes, respectively, so the gene expression of ADH2 and ALDH2 in the liver of mice in each group is similar to the level of ADH and ALDH (Figure 3), and ADH2 can promote the conversion of alcohol into acetaldehyde in the body, which is then further broken down into acetic acids by increasing the activity of ALDH2 [67]. Lf intervention significantly suppressed the hepatic *CYP2E1* expression compared with the Model group (*p* < 0.05) and significantly elevated the hepatic *ADH2* and *ALDH2* expression (*p* < 0.05), suggesting that Lf is helpful to promote the alcohol excretion. In addition, previous studies have confirmed that excessive alcohol consumption leads to oxidative stress, which accelerates the process of ALI [62]. In this study, Lf significantly elevated the hepatic *ADH2*, *ALDH2*, *Nrf2*, *CAT*, *HO-1*, *SOD1* and *GSH-Px* expression in mice with ALI. Among these factors, *Nrf2* (nuclear factor erythroid 2-related factor 2) serves as a key regulatory factor in both endogenous and exogenous antioxidant systems, while *iNOS* (inducible nitric oxide synthase) plays a critical role in inflammatory responses [68]. Under oxidative stress, Nrf2 dissociates from Keap1 and is transported to the nucleus to bind to ARE, thereby regulating the transcriptional activation of a series of antioxidant protection genes such as *HO-1*, *CAT*, *SOD1* and *GSH-Px* [17]. In addition, HO-1 acts as a critical protective enzyme that prevents inflammation-induced tissue injury [69]. Therefore, Nrf2/HO-1 signaling plays a critical role in alleviating a variety of liver-related diseases. These results suggest that Lf prevents alcohol-induced ALI by promoting alcohol metabolism and suppressing oxidative stress.

## 4. Conclusions

In present study, the improvement effects of *Limosilactobacillus fermentum* FZU501 (Lf) on ALI and its potential mechanisms were explored. The results demonstrated that oral administration of Lf may ameliorate the liver and intestinal barrier damage in mice with excessive alcohol consumption. The potential improvement mechanisms were illustrated by intestinal microbiomics, liver metabolomics and RT-qPCR. These hepatoprotective effects may be related to the regulation in gut microbiota composition, the modulation in liver metabonomic profile, and the adjustments in hepatic mRNA expressions of lipid metabolism, alcohol metabolism and antioxidant genes. The results of this study preliminarily explored the mitigation mechanism of Lf on ALI, and provided a scientific basis for the development of functional foods based on Lf. Future studies should focus on randomized controlled clinical trials of Lf intervention to further evaluate its improvement effect on intestinal microbiota homeostasis reconstruction, metabolic disorder regulation and clinical biochemical indicators in patients with alcoholic liver injury, so as to provide more convincing clinical evidence support for the development of evidence-based dietary intervention strategies.

## Data Availability

The original contributions presented in the study are included in the article; further inquiries can be directed to the corresponding author.

## Supplementary Materials

The following supporting information can be downloaded at: https://www.mdpi.com/article/10.3390/foods14061054/s1, Figure S1: Experimental feeding protocols for mice; Table S1: Main feed ingredient; Table S2: Nutritional guarantee value (per kilogram of feed); Table S3: Primer sequences for quantitative real-time PCR.

## Abbreviations

Lf, *Limosilactobacillus fermentum* FZU3103; ALI, alcoholic liver injury; ADH, alcohol dehydrogenase; ALDH, aldehyde dehydrogenase; SCFAs, short-chain fatty acids; MRS, Man–Rogosa–Sharpe; TC, total cholesterol; TG, triglyceride; LDL-C, low-density lipoprotein cholesterol; HDL-C, high-density lipoprotein cholesterol; AST, aspartate aminotransferase; ALT, alanine aminotransferase; CAT, catalase; SOD, superoxide dismutase; GSH-Px, glutathione peroxidase; PAHs, polycyclic aromatic hydrocarbons; H&E, hematoxylin–eosin; OTU, operation taxon units; PCA, principal component analysis; OPLS-DA, orthogonal partial least squares discriminant analysis; RT-qPCR, quantitative reverse transcription PCR; ROS, reactive oxygen species; ESI-, negative ion; ESI+, positive ion; AhR, aryl hydrocarbon receptor; LDLR, low-density lipoprotein receptor.

## References

[1] Ma Y., Xu Y., Tang K. (2025). Olfactory perception complexity induced by key odorants perceptual interactions of alcoholic beverages: Wine as a focus case example. Food Chem..

[2] Zheng Z., Liu K., Zhou Y., Debliquy M., Bittencourt C., Zhang C. (2025). A comprehensive overview of the principles and advances in electronic noses for the detection of alcoholic beverages. Trends Food Sci. Technol..

[3] World Health Organization (2024). Global Status Report on Alcohol and Health and Treatment of Substance Use Disorders.

[4] Chen H., Liu J., Peng S., Yang G., Cheng X., Chen L., Zhang H., Zhao Y., Yao P., Tang Y. (2023). Autophagy and Exosomes Coordinately Mediate Quercetin’s Protective Effects on Alcoholic Liver Disease. J. Nutr. Biochem..

[5] GBD 2016 Alcohol Collaborators (2018). Alcohol use and burden for 195 countries and territories, 1990–2016: A systematic analysis for the Global Burden of Disease Study 2016. Lancet.

[6] Wang W., Xu C., Wang Q., Hussain M.A., Wang C., Hou J., Jiang Z. (2023). Protective Effect of Polyphenols, Protein, Peptides, and Polysaccharides on Alcoholic Liver Disease: A Review of Research Status and Molecular Mechanisms. J. Agric. Food Chem..

[7] Wrzosek L., Ciocan D., Hugot C., Spatz M., Dupeux M., Houron C., Lievin-Le Moal V., Puchois V., Ferrere G., Trainel N. (2021). Microbiota Tryptophan Metabolism Induces Aryl Hydrocarbon Receptor Activation and Improves Alcohol-Induced Liver Injury. Gut.

[8] Albillos A., Gottardi A., Rescigno M. (2020). The gut-liver axis in liver disease: Pathophysiological basis for therapy. J. Hepatol..

[9] Xue L., He J., Gao N., Lu X., Li M., Wu X., Liu Z., Jin Y., Liu J., Xu J. (2017). Probiotics may delay the progression of nonalcoholic fatty liver disease by restoring the gut microbiota structure and improving intestinal endotoxemia. Sci. Rep..

[10] Ge S., Han J., Sun Q., Zhou Q., Ye Z., Li P., Gu Q. (2024). Research Progress on Improving the Freeze-Drying Resistance of Probiotics: A Review. Trends Food Sci. Technol..

[11] Plaza-Díaz J., Solís-Urra P., Rodríguez-Rodríguez F., Olivares-Arancibia J., Navarro-Oliveros M., Abadía-Molina F., Álvarez-Mercado A.I. (2020). The Gut Barrier, Intestinal Microbiota, and Liver Disease: Molecular Mechanisms and Strategies to Manage. Int. J. Mol. Sci..

[12] Ozen M., Piloquet H., Schaubeck M. (2023). *Limosilactobacillus fermentum* CECT5716: Clinical potential of a probiotic strain isolated from human milk. Nutrients.

[13] Naghmouchi K., Belguesmia Y., Bendali F., Spano G., Seal B., Drider D. (2020). *Lactobacillus fermentum*: A bacterial species with potential for food preservation and biomedical applications. Crit. Rev. Food Sci. Nutr..

[14] Kim B., Meng Z., Xu X., Baek S., Pathiraja D., Choi I., Oh S. (2023). Complete genome sequence of *Limosilactobacillus fermentum* JNU532 as a probiotic candidate for the functional food and feed supplements. J. Anim. Sci. Technol..

[15] Gillessen A., Schmidt H.J. (2020). Silymarin as Supportive Treatment in Liver Diseases: A Narrative Review. Adv. Ther..

[16] Wang M.-T., Guo W.-L., Yang Z.-Y., Chen F., Lin T.-T., Li W.-L., Lv X.-C., Rao P.-F., Ai L.-Z., Ni L. (2022). Intestinal microbiomics and liver metabolomics insights into the preventive effects of chromium (III)-enriched yeast on hyperlipidemia and hyperglycemia induced by high-fat and high-fructose diet. Curr. Res. Food Sci..

[17] Cao Y.-J., Huang Z.-R., You S.-Z., Guo W.-L., Zhang F., Liu B., Lv X.-C., Lin Z.-X., Liu P.-H. (2022). The protective effects of ganoderic acids from ganoderma lucidum fruiting body on alcoholic liver injury and intestinal microflora disturbance in mice with excessive alcohol intake. Foods.

[18] Dybiec J., Baran W., Dąbek B., Fularski P., Młynarska E., Radzioch E., Rysz J., Franczyk B. (2023). Advances in treatment of dyslipidemia. Int. J. Mol. Sci..

[19] Wang Z.-G., Wang X.-X., Wang Y.-L., Liu Y.-J., Wang X.-C., Song Y., Xue C.-H. (2023). Lipidomics Approach in Alcoholic Liver Disease Mice with Sphingolipid Metabolism Disorder: Alleviation Using Sea Cucumber Phospholipids. Food Biosci..

[20] Zhao L., Mehmood A., Yuan D., Usman M., Murtaza M.A., Yaqoob S., Wang C. (2021). Protective mechanism of edible food plants against alcoholic liver disease with special mention to polyphenolic compounds. Nutrients.

[21] Liu X., Hou R., Yan J., Xu K., Wu X., Lin W., Zheng M., Fu J. (2019). Purification and Characterization of Inonotus Hispidus Exopolysaccharide and Its Protective Effect on Acute Alcoholic Liver Injury in Mice. Int. J. Biol. Macromol..

[22] Li P., Jia J., Zhang D., Xie J., Xu X., Wei D. (2014). In vitro and in vivo antioxidant activities of a flavonoid isolated from celery (*Apium graveolens* L. var. dulce). Food Funct..

[23] Zhou D., Zhong J., Huang Y.-G., Cheng Y.-X. (2023). Effect of Free and Bound Polyphenols from Rosa Roxburghii Tratt Distiller’s Grains on Moderating Fecal Microbiota. Food Chem. X.

[24] Chambers E.S., Viardot A., Psichas A., Morrison D.J., Murphy K.G., Zac-Varghese S.E.K., MacDougall K., Preston T., Tedford C., Finlayson G.S. (2014). Effects of Targeted Delivery of Propionate to the Human Colon on Appetite Regulation, Body Weight Maintenance and Adiposity in Overweight Adults. Gut.

[25] Fairfield B., Schnabl B. (2020). Gut Dysbiosis as a Driver in Alcohol-Induced Liver Injury. JHEP Rep..

[26] Heimann E., Nyman M., Pålbrink A.-K., Lindkvist-Petersson K., Degerman E. (2016). Branched Short-Chain Fatty Acids Modulate Glucose and Lipid Metabolism in Primary Adipocytes. Adipocyte.

[27] Chae Y.-R., Lee Y.R., Kim Y.-S., Park H.-Y. (2024). Diet-Induced Gut Dysbiosis and Leaky Gut Syndrome. J. Microbiol. Biotechnol..

[28] Shen H., Zhou L., Zhang H., Yang Y., Jiang L., Wu D., Shu H., Zhang H., Xie L., Zhou K. (2024). Dietary Fiber Alleviates Alcoholic Liver Injury Via Bacteroides Acidifaciens and Subsequent Ammonia Detoxification. Cell Host Microbe.

[29] Bao T., He F., Zhang X., Zhu L., Wang Z., Lu H., Wang T., Li Y., Yang S., Wang H. (2020). Inulin exerts beneficial effects on non-alcoholic fatty liver disease via modulating gut microbiome and suppressing the lipopolysaccharide-toll-like receptor 4-mψ-nuclear factor-κb-nod-like receptor protein 3 pathway via gut-liver axis in mice. Front. Pharmacol..

[30] Rahman A.N.A., Abdelwarith A.A., Younis E.M., Rhouma N.R., Zaki H.T., Khalil S.S., El-Saber M.M., Davies S.J., El-Murr A., Ibrahim R.E. (2023). The Alleviative Effects of Green Synthesized Copper Oxide Nanoparticles against Oxidative Stress, Hepato-Renal Alterations, and Immune Suppression Induced by Staphylococcus Aureus Infection in Clarias Gariepinus. Aquac. Rep..

[31] Li L., Liu H., Yu J., Sun Z., Jiang M., Yu H., Wang C. (2023). Intestinal Microbiota and Metabolomics Reveal the Role of Auricularia delicate in Regulating Colitis-Associated Colorectal Cancer. Nutrients.

[32] Llorente C., Jepsen P., Inamine T., Wang L., Bluemel S., Wang H.J., Loomba R., Bajaj J.S., Schubert M.L., Sikaroodi M. (2017). Gastric Acid Suppression Promotes Alcoholic Liver Disease by Inducing Overgrowth of Intestinal Enterococcus. Nat. Commun..

[33] Tian L., Zhao R., Xu X., Zhou Z., Xu X., Luo D., Zhou Z., Liu Y., Kushmaro A., Marks R. (2022). Modulatory Effects of Lactiplantibacillus Plantarum on Chronic Metabolic Diseases. Food Sci. Hum. Wellness.

[34] Lv X.-C., Wu Q., Yuan Y.-J., Li L., Guo W.-L., Lin X.-B., Huang Z.-R., Rao P.-F., Ai L.-Z., Ni L. (2022). Organic Chromium Derived from the Chelation of Ganoderma Lucidum Polysaccharide and Chromium (Iii) Alleviates Metabolic Syndromes and Intestinal Microbiota Dysbiosis Induced by High-Fat and High-Fructose Diet. Int. J. Biol. Macromol..

[35] Verbrugghe P., Brynjólfsson J., Jing X., Björck I., Hållenius F., Nilsson A. (2021). Evaluation of Hypoglycemic Effect, Safety and Immunomodulation of Prevotella Copri in Mice. Sci. Rep..

[36] Sun X.-Q., Shi J.-J., Kong L.-Y., Shen Q.-Y., Zeng X.-Q., Wu Z., Guo Y.-X., Pan D.-D. (2022). Recent Insights into the Hepatoprotective Effects of Lactic Acid Bacteria in Alcoholic Liver Disease. Trends Food Sci. Technol..

[37] Duan W., Liu F., Ren Y., Zhang X., Shi J., Xue Y., Xu Z., Geng Y. (2024). Differences in the Ability of Lactic Acid Bacteria To Prevent Acute Alcohol-Induced Liver Injury via the Gut Microbiota–Bile Acid–Liver Axis. J. Agric. Food Chem..

[38] Sinha S.R., Haileselassie Y., Nguyen L.P., Tropini C., Wang M., Becker L.S., Sim D., Jarr K., Spear E.T., Singh G. (2020). Dysbiosis-induced secondary bile acid deficiency promotes intestinal inflammation. Cell Host Microbe.

[39] Ziętak M., Kovatcheva-Datchary P., Markiewicz L.H., Ståhlman M., Kozak L.P., Bäckhed F. (2016). Altered Microbiota Contributes to Reduced Diet-Induced Obesity Upon Cold Exposure. Cell Metab..

[40] Serena C., Ceperuelo-Mallafré V., Keiran N., Queipo-Ortuño M.I., Bernal R., Gomez-Huelgas R., Urpi-Sarda M., Sabater M., Pérez-Brocal V., Andrés-Lacueva C. (2018). Elevated Circulating Levels of Succinate in Human Obesity Are Linked to Specific Gut Microbiota. ISME J..

[41] Zou Y., Ding W., Wu Y., Chen T., Ruan Z. (2023). Puerarin Alleviates Inflammation and Pathological Damage in Colitis Mice by Regulating Metabolism and Gut Microbiota. Front. Microbiol..

[42] Mialon N., Roig B., Capodanno E., Cadiere A. (2023). Untargeted metabolomic approaches in food authenticity: A review that showcases biomarkers. Food Chem..

[43] Zhong P., Wei X., Li X., Wei X., Wu S., Huang W., Koidis A., Xu Z., Lei H. (2022). Untargeted metabolomics by liquid chromatography-mass spectrometry for food authentication: A review. Compr. Rev. Food Sci. Food Saf..

[44] Vasilchenko A.S., Gurina E.V., Drozdov K.A., Vershinin N.A., Kravchenko S.V., Vasilchenko A.V. (2023). Exploring the Antibacterial Action of Gliotoxin: Does It Induce Oxidative Stress or Protein Damage?. Biochimie.

[45] Zheng R., Michaëlsson K., Fall T., Elmståhl S., Lind L. (2023). The Metabolomic Profiling of Total Fat and Fat Distribution in a Multi-Cohort Study of Women and Men. Sci. Rep..

[46] Tao L.-P., Li X., Zhao M.-Z., Shi J.-R., Ji S.-Q., Jiang W.-Y., Liang Q.-J., Lei Y.-H., Zhou Y.-Y., Cheng R. (2021). Chrysene, a Four-Ring Polycyclic Aromatic Hydrocarbon, Induces Hepatotoxicity in Mice by Activation of the Aryl Hydrocarbon Receptor (Ahr). Chemosphere.

[47] Lu Y., Huang C., Gao L., Xu Y., Chia S.E., Chen S., Li N., Yu K., Ling Q., Cheng Q. (2015). Identification of serum biomarkers associated with hepatitis B virus-related hepatocellular carcinoma and liver cirrhosis using mass-spectrometry-based metabolomics. Metabolomics.

[48] Samanthula K.S., Bairi A.G., Mahendra Kumar C.B. (2021). Muco-Adhesive Buccal Tablets of Candesartan Cilexetil for Oral Delivery: Preparation, in-Vitro and Ex-Vivo Evaluation. J. Drug Deliv. Ther..

[49] Peng W.-Q., Xiao G., Li B.-Y., Guo Y.-Y., Guo L., Tang Q.-Q. (2021). L-Theanine Activates the Browning of White Adipose Tissue through the Ampk/{Alpha}-Ketoglutarate/Prdm16 Axis and Ameliorates Diet-Induced Obesity in Mice. Diabetes.

[50] Zhao X., Wang L., Zhang H., Zhang D., Zhang Z., Zhang J. (2017). Protective Effect of Artemisinin on Chronic Alcohol Induced-Liver Damage in Mice. Environ. Toxicol. Pharmacol..

[51] Tashita C., Hoshi M., Hirata A., Nakamoto K., Ando T., Hattori T., Yamamoto Y., Tezuka H., Tomita H., Hara A. (2020). Kynurenine Plays an Immunosuppressive Role in 2,4,6-Trinitrobenzene Sulfate-Induced Colitis in Mice. World J. Gastroenterol..

[52] Matsuo H., Kondo Y., Kawasaki T., Tokuyama S., Imamura N. (2015). Borrelidin Isolated from *Streptomyces* Sp. Inhibited Adipocyte Differentiation in 3t3-L1 Cells Via Several Factors Including Gata-Binding Protein 3. Biol. Pharm. Bull..

[53] Kim S.-J., Ise H., Kim E., Goto M., Akaike T., Chung B.H. (2013). Imaging and Therapy of Liver Fibrosis Using Bioreducible Polyethylenimine/Sirna Complexes Conjugated with N-Acetylglucosamine as a Targeting Moiety. Biomaterials.

[54] Beaumont M., Neyrinck A.M., Olivares M., Rodriguez J., de Rocca Serra A., Roumain M., Bindels L.B., Cani P.D., Evenepoel P., Muccioli G.G. (2018). The Gut Microbiota Metabolite Indole Alleviates Liver Inflammation in Mice. FASEB J..

[55] Zhang Y., Jia H., Jin Y., Liu N., Chen J., Yang Y., Dai Z., Wang C., Wu G., Wu Z. (2020). Glycine Attenuates Lps-Induced Apoptosis and Inflammatory Cell Infiltration in Mouse Liver. J. Nutr..

[56] Nakatake R., Okumura T., Miki H., Ueyama Y., Tsuda T., Nakamura Y., Tokuhara K., Kaibori M., Nishizawa M., Kwon A.H. (2014). Lb014-Sun: Glutamic Acid Has a Liver-Protective Effect through the Suppression of Inducible Nitric Oxide Synthase. Clin. Nutr..

[57] Lewerenz H.J., Plass R., Bleyl D.W., Macholz R. (1988). Short-Term Toxicity Study of Allyl Isothiocyanate in Rats. Food/Nahrung.

[58] Thomas C.E., Luu H.N., Wang R., Xie G., Adams-Haduch J., Jin A., Koh W.-P., Jia W., Behari J., Yuan J.-M. (2021). Association between Pre-Diagnostic Serum Bile Acids and Hepatocellular Carcinoma: The Singapore Chinese Health Study. Cancers.

[59] Bjøm K., Gjerstad L., Bentdal Ø., Osnes S., Schrumpf E. (1998). Topiramate and Fulminant Liver Failure. Lancet.

[60] Zhong S., Li L., Liang N., Zhang L., Xu X., Chen S., Yin H. (2021). Acetaldehyde Dehydrogenase 2 Regulates Hmg-Coa Reductase Stability and Cholesterol Synthesis in the Liver. Redox Biol..

[61] Lim M.Y.C., Ho H.K. (2024). Pharmacological modulation of cholesterol 7α-hydroxylase (CYP7A1) as a therapeutic strategy for hypercholesterolemia. Biochem. Pharmacol..

[62] Wu L., Zhou K., Yang Z., Li J., Chen G., Wu Q., Lv X., Hu W., Rao P., Ai L. (2022). Monascuspiloin from Monascus-Fermented Red Mold Rice Alleviates Alcoholic Liver Injury and Modulates Intestinal Microbiota. Foods.

[63] Zhu H., Zhao T., Zhao S., Yang S., Jiang K., Li S., Kang Y., Yang Z., Shen J., Shen S. (2024). O-Glcnacylation Promotes the Progression of Nonalcoholic Fatty Liver Disease by Upregulating the Expression and Function of Cd36. Metabolism.

[64] Quan J., Bode A.M., Luo X. (2021). Acsl Family: The Regulatory Mechanisms and Therapeutic Implications in Cancer. Eur. J. Pharmacol..

[65] Tahri-Joutey M., Andreoletti P., Surapureddi S., Nasser B., Cherkaoui-Malki M., Latruffe N. (2021). Mechanisms mediating the regulation of peroxisomal fatty acid beta-oxidation by PPARα. Int. J. Mol. Sci..

[66] Carrasco D., Carrasco C., Souza-Mello V., Sandoval C. (2021). Effectiveness of antioxidant treatments on cytochrome P450 2E1 (CYP2E1) activity after alcohol exposure in humans and in vitro models: A systematic review. Int. J. Food Prop..

[67] Yan T., Zhang Y., Lu H., Zhao J., Wen C., Song S., Ai C., Yang J. (2024). The Protective Effect of Enteromorpha Prolifera Polysaccharide on Alcoholic Liver Injury in C57bl/6 Mice. Int. J. Biol. Macromol..

[68] Liu S., Zhao Y., Xu X., Wang M., Tao X., Xu H. (2023). Lactiplantibacillus Plantarum P101 Alleviates Alcoholic Liver Injury by Modulating the Nrf2/Ho-1 Pathway in Mice. J. Appl. Microbiol..

[69] Wang R.Q., Nan Y.M., Wu W.J., Kong L.B., Han F., Zhao S.X., Kong L., Yu J. (2011). Induction of heme oxygenase-1 protects against nutritional fibrosing steatohepatitis in mice. Lipids Health Dis..

