# Supplementary material for: The Protective Effect of Limosilactobacillus fermentum FZU501 Against Alcohol-Induced Liver Injury in Mice via Gut Microbiota–Liver Axis
Source: Foods. 2025 Mar 19;14(6):1054. doi: 10.3390/foods14061054 (PMC11942275; doi:10.3390/foods14061054)
Supplement: Supplementary file 1 [file foods-14-01054-s001.zip › foods-3510462-supplementary.pdf]

# Supplementary materials

Table S1. Main feed ingredient

| Nutrient sources | Ingredients |
|---|---|
| Protein source | Peruvian red fish meal, high quality soybean meal |
| Fat source | Food grade soybean oil, sunflower oil |
| Carbohydrate source | Yellow corn, sub-flour, bran |
| Others | Vitamins, minerals, amino acids |

Table S2. Nutritional guarantee value (per kilogram of feed)

| Nutritional ingredient | Content | Nutritional ingredient | Content |
|---|---|---|---|
| Crude protein | ≥180 g | Calcium (Ca) | 10-18 g |
| Crude fat | ≥40 g | Phosphorus (P) | 6-12 g |
| Moisture | ≤100 g | Lysine | ≥8.2 g |
| Crude ash | ≤80 g | Cystine | ≥5.3 g |
| Coarse fibre | ≤50 g | Vitamin E | ≥60 IU |

Table S3. Primer sequences for quantitative real-time PCR.

| Gene | Forward primer (5′−3′) | Reverse primer (5′−3′) |
|---|---|---|
| *Ldlr* | ATGCTGGAGATAGAGTGGAGT | CCGCCAAGATCAAGAAA |
| *Cyp71a* | CCTTGGGACGTTTTCCTGCT | GCGCTCTTTGATTTAGGA |
| *Cpt-1* | TCCATGCATACCAAAGTGGA | TGGTAGGAGAGCAGCAC |
| *Acox1* | GCCTGCTGTGTGGGTATGTCA | GTCATGGGCGGGTGCAT |
| *Acsl1* | CACTTCTTGCCTCGTTCCAC | GTCGTCCCGCTCTATGAC |
| *Ppar-a* | TGCCTTCCCTGTGAACTGAC | TGGGGAGAGAGGACAGA |
| *CYP2E1* | CCAACTCTGGACTCCCTTTTA | ACGCCTTGAAATAGTCAC |
| *ADH2* | AACGGTGAGAAGTTCCCAAA | ACGACCCCCAGCCTAATA |
| *ALDH2* | ATCCTCGGCTACATCAAATCG | GTCTTTTACGTCCCCGAA |
| *Nrf2* | CCGGGAKCAAKCAGAKA | ACGTTGTCCCCATTTTTG |
| *CAT* | TCACCCACGATATCACCAGA | AGCTGAGCCTGACTCTC |
| *HO-1* | AACAAGCAAKCCCAGTCTAT | AGGTAGCGGGTATATGCG |
| *SOD1* | TTGGCCGTACAA GGTGG | CGCAATCCCAATCACTCC |
| *GSH-Px* | GGGACCCTGAGACTTAGAGC | AATCCGTACTAGCGCTCA |
| *Cd36* | ACTTGGGATTGGAGTGGTGAT | GGATACCTGCAGTTTGAG |
| *HMGCR* | TGCTGGTGCTATCAAAGG | GCAGATGGGATGACTCG |
| Mouse 18S | AGTCCCTGCCCTTTGTACACA | CGATCCCAGGGCCTCACT |

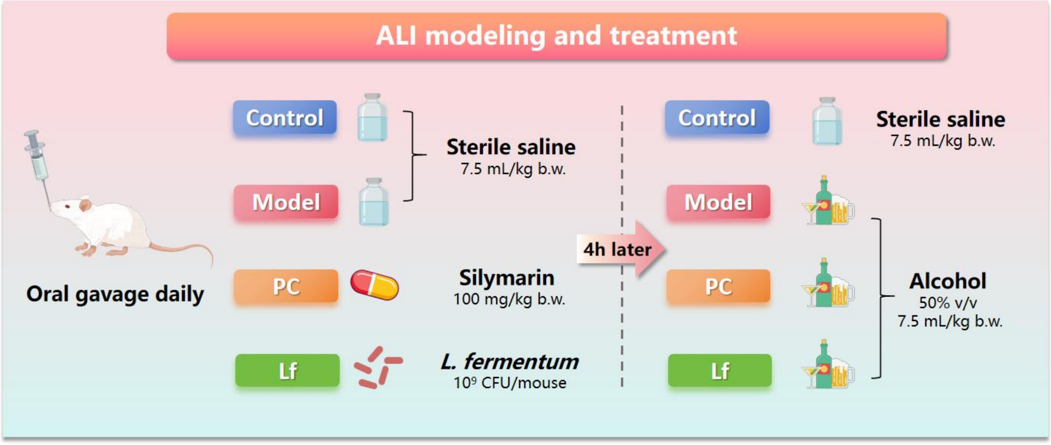


Figure S1. Experimental feeding protocols for mice.
